# An examination of anhedonia as a predictor of response with transcranial magnetic stimulation treatment for youth with depression^☆^

**DOI:** 10.1016/j.xjmad.2025.100147

**Published:** 2025-09-03

**Authors:** Jacquelin C. Hecker, Paul A. Nakonezny, Cicek N. Bakir, Irem Azamet, Magdalena Romanowicz, Julia Shekunov, Jennifer L. Vande Voort, J. Luis Lujan, Paul E. Croarkin

**Affiliations:** a Center for Clinical and Translational Science, Mayo Clinic Graduate School of Biomedical Sciences, Rochester, MN 55905, USA; b Department of Population and Data Sciences, UT Southwestern Medical Center, Dallas, TX 75390, USA; c Mayo Clinic School of Graduate Medical Education, Mayo Clinic College of Medicine and Science, Rochester, MN 55905, USA; d Department of Psychiatry and Psychology, Mayo Clinic, Rochester, MN 55905, USA; e Department of Neurologic Surgery and Department of Physiology and Biomedical Engineering, Mayo Clinic, Rochester, MN 55905, USA

**Keywords:** Adolescent, Anhedonia, Major depressive disorder, Transcranial magnetic stimulation, Treatment response

## Abstract

This exploratory study examined the relationship between the change in anhedonia symptoms and treatment response in adolescents receiving 1 Hz or 10 Hz transcranial magnetic stimulation (TMS) treatment for major depressive disorder (MDD). Participants were aged 12–18 years, had a depressive symptom score of 40 or higher on the Children’s Depressed Rating Scale-Revised (CDRS-R), and were randomized to either the 1 Hz (n = 22) or 10 Hz (n = 19) group for 30 daily TMS treatments over the left dorsolateral prefrontal cortex (LDLPFC). Anhedonia was measured using items from the Beck Depression Inventory-II and CDRS-R. Treatment outcome was assessed with the Clinical Global Impressions-Improvement scales (CGI-I). Logistic regression was used to estimate the odds of CGI-I treatment response from the change in anhedonia symptoms (baseline to week 6), and a linear mixed model of repeated measures analyzed the change in anhedonia (baseline, weeks 4 and 6) compared between the TMS treatment groups over the 6-week study period. TMS stimulus frequency did not affect the change in anhedonia and CGI-I response. There was a significant inverse relationship concerning the change in anhedonia symptoms and CGI-I response for both the 10 Hz (p = 0.0109, δ= −0.8581, SE= 0.3371) and 1 Hz (p = 0.0277, δ= −1.1017, SE=0.5003) groups. Thus, as anhedonia symptoms improved over the 6 weeks, the probability of CGI-I response at week 6 increased for both the 1 Hz and 10 Hz TMS groups. An adjusted least squares for anhedonia revealed a significant improvement in anhedonia for TMS groups (10 Hz, p < 0.0001, *d=*0.9032; 1 Hz, p < 0.0001, *d*=0.8536). Future studies of anhedonia may inform precision TMS treatments for youth.

## Highlights

• Anhedonia is a key depressive symptom that may predict treatment outcomes.
• Anhedonia in adolescents receiving TMS is understudied.
• Changes in anhedonia had a relationship with TMS treatment response.
• Anhedonia improved with low or high frequency TMS.

## Graphical Abstract

**Figure:**
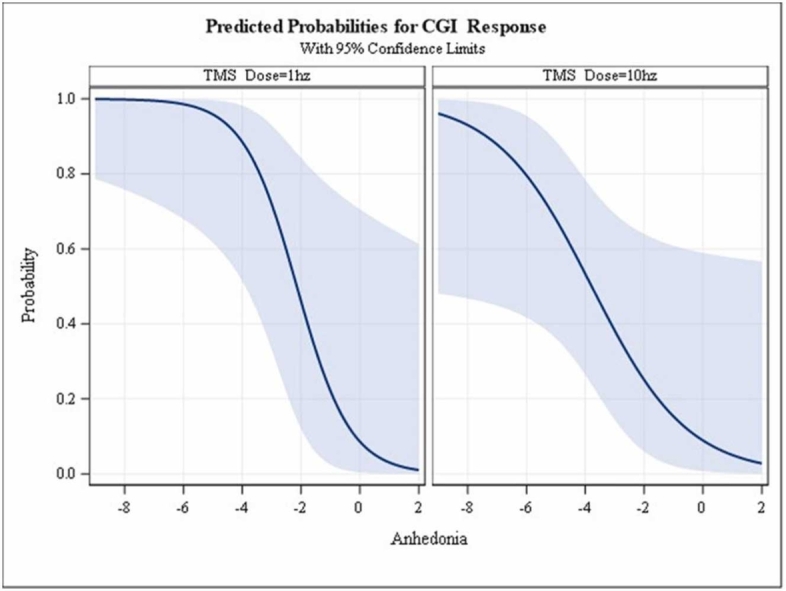

## 1 Introduction

Experience with transcranial magnetic stimulation (TMS) as a treatment for adolescents with major depressive disorder (MDD) continues to develop. TMS received FDA clearance in 2024 for the adjunctive treatment of MDD for patients aged 15–21 years. However, prior research with TMS treatments for adolescents with MDD suggests that the clinical effect of TMS may be modest [1]. There is a pressing need to improve clinical outcomes with TMS treatment though the identification of baseline neurobiological [2] and clinical moderators, such as anhedonia [3].

Gamma-aminobutyric acid (GABA) and glutamate neurotransmission have been considered in the pathophysiology of mood disorders and mechanism of TMS [4], [5]. Prior research focused on adolescents suggests that anhedonia is associated with reduced GABA levels in the anterior cingulate cortex, a key region involved in reward processing [6]. Individuals diagnosed with mood disorders consistently exhibit reduced GABA concentrations in both plasma and cerebrospinal fluid [7]. Similarly, preclinical models of depression demonstrate decreased GABA levels, with GABA agonist administration shown to reverse depressive-like behaviors. Notably, low-frequency transcranial magnetic stimulation (TMS) at 1 Hz is thought to enhance GABAergic function and may represent a promising therapeutic intervention for adolescents with MDD and anhedonia [8], [9].

Anhedonia is a core symptom and diagnostic hallmark of major depressive episodes. Among adolescents, the presence of anhedonia is strongly associated with suicide-related outcomes and is linked to distinct neurobiological differences in reward processing networks compared to peers without anhedonia [10], [11]. The diagnostic criteria for MDD, as outlined in the *Diagnostic and Statistical Manual of Mental Disorders*, Third Edition, Revised (DSM-III-R), allow for the substitution of irritability in place of depressed mood or anhedonia for diagnosing depression in adolescents [12]. However, subsequent research focused on adolescents demonstrated that anhedonia severity is more robustly associated with overall illness severity, episode duration, and the number of depressive episodes than irritability alone [13].

Anhedonia has also been identified as a potential moderator of clinical outcomes in both adolescent and adult populations. In adults with depression, higher baseline levels of anhedonia have been associated with poorer treatment outcomes, including prolonged remission and recovery times. Notably, individuals with elevated anhedonia responded more favorably to combining cognitive therapy and pharmacological treatment, whereas those with lower levels of anhedonia showed no significant difference in response to combined therapies versus pharmacological treatment alone [14]. Similarly, in adolescents with treatment resistant depression, greater baseline anhedonia predicted a longer time to remission and fewer depression-free days during recovery [15].

Anhedonia warrants ongoing consideration as a clinical marker in treatment planning for adolescents with MDD. Its presence may help identify adolescents with MDD who are less likely to respond to conventional pharmacotherapy and may benefit from alternative or adjunctive interventions such as TMS, ketamine or other emerging neurotherapeutics. Furthermore, the severity of anhedonia may determine parameters such as high or low frequency during the TMS delivery of depression treatment. With these considerations in mind, this study examined the relationship between the change in anhedonia symptoms and treatment response in adolescents with MDD undergoing 6 weeks of 1 Hz or 10 Hz TMS treatment. It was hypothesized that change in anhedonia symptoms would be associated with treatment response and that there would be differential effects with 1 Hz and 10 Hz dosing.

## 2 Method

### 2.1 Participants

This was an exploratory analysis of a double-blind, randomized trial of 1 Hz and 10 Hz repetitive TMS for adolescents with MDD. The protocol and parent clinical trial have been published elsewhere [2], [16]. Participants in this analysis were 12–18 years of age and had a score of 40 or greater on the Children’s Depression Rating Scale-Revised (CDRS-R). Participants were randomly assigned to receive 30 daily sessions of either 1 Hz or 10 Hz TMS at 120 % of the resting motor threshold, targeting the left dorsolateral prefrontal cortex (LDLPFC). Individuals who completed less than one week of treatment were excluded from all analyses due to the absence of follow-up clinical assessments. The Mayo Clinic Institutional Review Board approved the study. Informed assent and consent were obtained from all participants and their parents or guardians prior to any research procedures. The trial was registered at ClinicalTrials.gov (identifier: NCT03363919).

### 2.2 Assessments

For the present exploratory study, outcomes were assessed with the clinician-rated Clinical Global Impressions-Severity (CGI-S), which was measured at baseline, and the clinician-rated Clinical Global Impressions - Improvement (CGI-I), which was measured at posttreatment (week 6). Based on prior work, the Anhedonia scale was operationally defined as composite score of items 4 and 12 on the Beck Depression Inventory-II (self-report inventory) and item 2 from the CDRS-R (clinician-rated) and measured at baseline, week 4, and posttreatment (week 6) [17]. A score of 1 or 2 on the CGI-I scale at week 6 was classified as response to TMS treatment. The CGI was used for the outcome measure to mitigate collinearity as anhedonia symptom severity was derived from the CDRS-R and Beck Depress Inventory-II.

### 2.3 Statistical analysis

Logistic regression, with penalized maximum likelihood estimation along with Firth’s bias correction (to limit bias due to small samples), was implemented to estimate the odds of CGI-I treatment response at week 6 (as the binary outcome) from the change in Anhedonia symptoms (baseline to week 6). The model contained fixed effects terms for TMS treatment, change in anhedonia symptoms, and TMS treatment x change in anhedonia symptoms interaction. Baseline CGI-S, age, sex at birth, and number of previously failed medication trials were included as covariates in the model. The log odds were reported along with the predicted probabilities of CGI treatment response at levels of change in anhedonia symptoms. The area under the ROC curve (AUC) was reported to demonstrate discriminative ability of the logistic regression model.

The change in anhedonia over the 6-week study period (baseline, weeks 4 and 6) was compared between the TMS treatment groups (1 Hz vs. 10 Hz TMS) using a linear mixed model analysis of repeated measures. The mixed model contained fixed effects terms for TMS treatment, time, and TMS treatment x time interaction. Baseline CGI-S, age, sex, and number of previously failed medication trials were included as covariates in the model. Restricted maximum likelihood estimation along with Type 3 tests of fixed effects were used with the Kenward-Roger correction applied to the autoregressive (AR1) covariance structure. We modeled the AR(1) correlation structure in the residuals across time within each subject using the random statement in PROC GLIMMIX in SAS. This allowed us to account for variability over time that was not captured by fixed effects. Least squares (LS) means were estimated as part of the mixed model to interpret the TMS group effect (LS mean difference between groups). Simple TMS group effects at each time period as well as within-group change over the 6 weeks were also assessed. Cohen’s *d* was calculated and interpreted as the effect size estimator.

Statistical analyses were conducted using SAS software, version 9.4. The level of significance was set at α = 0.05 (two-tailed).

## 3 Results

### 3.1 Participant characteristics

A full description of the study participants' characteristics can be found elsewhere [2]. Of the 41 youth, 60.98 % were female sex, 82.92 % were white, and the mean age was 15.78 ± 1.51 years (range 12–18 years). Of the 36 youth who completed all 6 weeks of treatment, 61.11 % showed a CGI-I rated response. For those in the 1 Hz TMS group, 13/19 (68.42 %) of youth responded, while those in the 10 Hz TMS group, 9/17 (52.94 %) of youth responded. The 10 Hz and 1 Hz TMS treatment groups did not differ on any of the demographic or clinical characteristics.

### 3.2 CGI-I treatment response

**Fig. 1:**
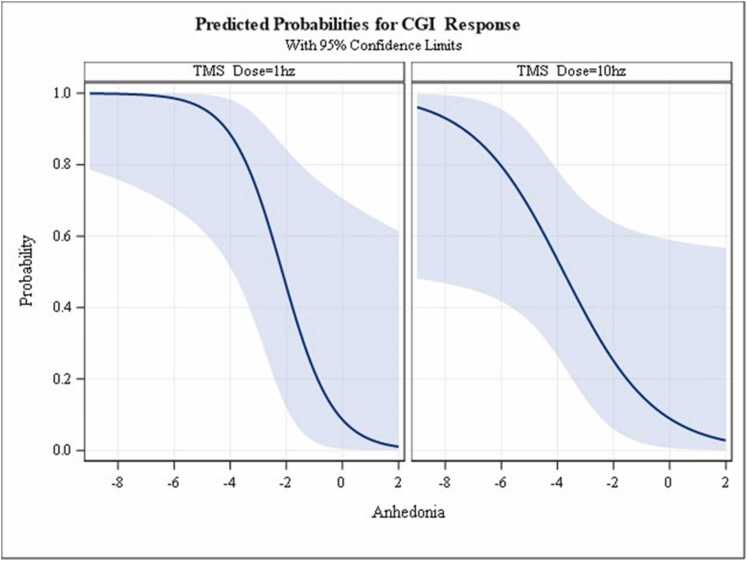
Predicted probabilities of response based on change in anhedonia. Logistic regression results. CGI-I Response at week 6 for TMS treatment (1 Hz vs 10 Hz) interacted with the change in anhedonia symptoms from baseline to week 6. Anhedonia = week 6 minus baseline. Probability of treatment response was adjusted for baseline CGI severity, age, sex, and number of previous failed medications. Note. Change in anhedonia symptom score: A negative [-] change score value = improvement in symptoms (e.g., −1 means improvement by 1 scale point, −2 means improvement by 2 scale points…, and so forth), 0 =no improvement, and a positive [+ ] change score value =worsening anhedonia symptoms.

The results from the logistic regression (AUC=0.948) revealed a significant negative (inverse) relationship between the change in anhedonia symptoms and CGI-I response for the 10 Hz TMS group (δ log odds = −0.8581, SE = 0.3371, 95 % CI −1.5187 to −0.1975; p = 0.0109) and for the 1 Hz TMS group (δ log odds = −1.1017, SE = 0.5003, 95 % CI −2.0821 to −0.1212; p = 0.0277). What this means, as anhedonia symptoms improved over the 6 weeks, the probability of CGI-I response at week 6 increased for both the 1 Hz and 10 Hz TMS groups. However, there was no significant interaction between TMS treatment group and change in anhedonia symptoms on CGI-I response (p = 0.3204). Thus, TMS stimulus frequency (10 Hz vs. 1 Hz) did not moderate the relationship between the change in anhedonia symptoms and CGI-I response. Although this suggests that improvement in anhedonia symptoms over 6 weeks was associated with CGI-I response regardless of TMS frequency, examination of Fig. 1 shows that a greater probability of treatment response was observed at all levels of improvement in anhedonia symptoms, especially at lower levels of improvement (e.g., −2 to −4) for the 1 Hz TMS frequency versus the 10 Hz TMS frequency.

### 3.3 Improvements in anhedonia

**Fig. 2:**
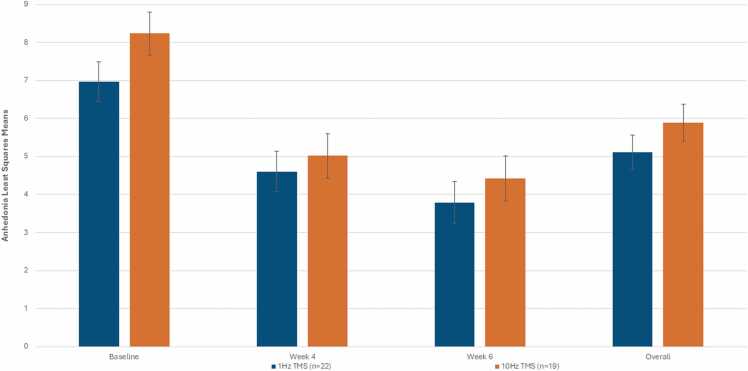
Change in anhedonia across TMS treatments**.** Mixed model results. Anhedonia symptoms over the 6-week study period by TMS treatment (1 Hz vs. 10 Hz TMS). Least squares means (LSM) are adjusted for baseline CGI-Severity, age, sex, and number of previous failed medications. Overall = timed-average LSM across baseline and weeks 4, 6. There were no significant between-subject TMS group effects overall or at any individual week However, for the within-group simple effects, the pattern of the adjusted anhedonia least squares means revealed a significant change (or improvement) in anhedonia symptoms across the 6 week trial for the 1 Hz TMS group (p < 0.0001, *d*=0.853) an*d* for the 10 Hz TMS group (p < 0.0001, *d*=0.903).

The mixed model repeated measures analysis revealed that there was no significant TMS treatment group by period interaction effect (p = 0.4411) as well as no significant main effect of TMS treatment group (p = 0.2559), but there was a significant period effect (p < 0.0001) on change in anhedonia symptoms over the 6-week trial. The pattern of the adjusted anhedonia least squares revealed a significant change (or improvement) in anhedonia across the 6-week trial for the 1 Hz TMS group (p < 0.0001, *d*=0.8536) and for the 10 Hz TMS group (p < 0.0001, *d*=0.9032; Fig. 2). However, although not significant, the pattern of the overall least squares TMS treatment group means over the 6-week trial showed that adjusted anhedonia was lower for the 1 Hz TMS group vs. the 10 Hz TMS group [5.121 (SE = 0.450) vs. 5.894 (SE = 0.486), p = 0.2559; *d*= 0.3804, Fig. 2]. The simple TMS treatment group effects at the individual weeks also revealed no significant TMS group differences (p-values<0.4349; Fig. 2).

## 4 Discussion

This was an exploratory study examining how the change in anhedonia symptoms is related to TMS treatment response for adolescents with MDD. Our results show that as anhedonia symptoms improve, the probability of CGI-I treatment response increases. However, there was no difference when investigating specific frequencies (1 Hz vs. 10 Hz) and their effects on the improvement of this relationship. The original study chose the 1 Hz and 10 Hz frequency parameters based on the conceptualization that lower frequencies (1 Hz) inhibit neural activity while high frequencies (10 Hz) have an excitatory effect [2]. Although prior research on left ventral striatal reward responses conceptually supports the idea that high-frequency TMS would more effectively reduce anhedonia in youth compared to low-frequency stimulation, our findings did not support this theory [18]. Although, there was a significant period effect on change in anhedonia symptoms over the duration of the trial, as well as a significant change in anhedonia, overall, within the 1 Hz and 10 Hz TMS groups. Anhedonia scores were lower in the group that received TMS at 1 Hz compared to the group that received 10 Hz stimulation. However, these differences were not significant. Current literature studying TMS’s effects on anhedonia have not extensively researched frequency effects, as most experiments use depression protocols that only focus on one frequency (or do not seek to compare). The present findings add to literature that seeks to understand how anhedonia affects TMS treatment outcome and will inform future TMS protocols.

Multiple prior studies have evaluated the potential impact of anhedonia on behavioral outcomes, such as suicidal ideation and other MDD-related outcomes [19], [20, [21]. This work includes efforts examining how anhedonia may moderate treatment outcomes through clinical interventions such as SSRIs and cognitive therapy [14], [15]. However, research focused on the impact of anhedonia on the clinical course of youth is limited, and requires further investigation into other treatment techniques, transdiagnostic considerations, and patient populations.

Recent work found marked changes in depression, anhedonia, and suicidal ideation because of TMS treatment [3]. In contrast, results from a different study by Fukuda and colleagues found that while TMS improved anhedonia and depressive symptoms, anhedonia severity at baseline had no effect on clinical outcomes [22]. The present study sought to help fill this gap by examining how anhedonia acts as a moderator of TMS treatment response in adolescents with MDD. Although there were limited differences between the 1 Hz and 10 Hz treatment arms, our results showed that anhedonia was reduced following TMS treatment and laid the foundation for future research.

The improvement in CGI-I scores and anhedonia severity under the 1 Hz and 10 Hz treatment protocols in our study highlights the importance of alternative therapeutic research for MDD. A separate study evaluating a novel TMS approach to modulate reward circuitry found that those who had lower baseline reward function had greater treatment efficacy [23]. This could be translated to future studies seeking to modulate reward circuitry using individualized neuromodulation to treat depressive and anhedonia-like symptoms. Although our study had no differences in outcome between the two frequency groups, the results in combination with other current literature beg for further clarification of differences in TMS treatment protocols and various biomarkers or assessments that can be used to tailor treatment.

The present study is one of the first of its kind by investigating anhedonia and its effects on MDD TMS treatment outcome, stratified by TMS frequency treatment groups. There are limitations that must be acknowledged. First, the study was performed on a small and homogenous patient cohort, which limits statistical power and generalizability. For this reason, further research should be performed with larger, more heterogeneous patient cohorts. Second, anhedonia was assessed through a composite score using the Beck Depression Inventory-II and Children’s Depression Rating Scale Revised, without additional neural measurements such as fMRI, which may limit accuracy of the results. To mitigate this, future studies should rely on anhedonia-specific scales such as the Snaith-Hamilton Pleasure Scale, the Pleasure Scale, or the Temporal Experience of Pleasure Scale [24], [25], [26] and neural confirmations, where appropriate. Finally, this study did not include a sham-control group to compare placebo effects. There was no between-group differences among TMS frequencies, changes over time in anhedonia and depression symptoms may have arisen from sources other than TMS intervention (such as expectancy effects or regression to the mean).

## 5 Conclusion

This study evaluated anhedonia scores and adolescent major depressive disorder improvement over 6 weeks with either 1 Hz or 10 Hz TMS. The results demonstrate that as anhedonia symptoms improve, the probability of CGI-I response increases over the treatment period in both groups, with no specific effect of frequency (1 Hz. vs. 10 Hz). Overall, both TMS treatment groups had significantly marked improvement of anhedonia symptoms and CGI-I scores from baseline to the end of the trial. Our results suggest that TMS treatment may address anhedonic symptoms of MDD and provide a foundation for optimizing TMS treatment protocols for adolescents with MDD.

## Ethics approval

Prior to any research activities, an investigational device exemption was obtained from the FDA (G170212), and the study was reviewed and approved by the Mayo Clinic Institutional Review Board, study number #17–004958, PI: Paul E Croarkin, DO, MS.

## Funding

This project was funded by the 10.13039/100000025National Institute of Mental Health (NIMH) under Award Number R01MH113700. The content is solely the responsibility of the authors and does not necessarily represent the official views of the National Institutes of Health or NIMH. Neuronetics, Inc., provided device and equipment support through a grant-in-kind but had no role in the study design, study execution, data analyses, data interpretation, or drafting of the manuscript.

Paul A. Nakonezny, PhD, served as the statistical expert for this research.

The authors express their appreciation to the families who participated in the study.

## CRediT authorship contribution statement

**JCH:** Conceptualization, Writing-Original Draft, Writing-Review & Editing, Visualization. **PAN:** Conceptualization, Formal Analysis, Methodology, Investigation, Writing-Review & Editing, Visualization Supervision, Project Administration, Funding Acquisition. **CNB:** Conceptualization, Writing-Review & Editing, Visualization. **IA:** Conceptualization, Writing-Review & Editing, Visualization. **MR:** Conceptualization, Investigation, Writing-Review & Editing. **JS:** Conceptualization, Investigation, Writing-Review & Editing. **JVV:** Conceptualization, Investigation, Writing-Review & Editing, Supervision, Project Administration. **JLL:** Conceptualization, Writing-Original Draft, Writing-Review & Editing, Supervision, Funding Acquisition. **PEC:** Conceptualization, Methodology, Investigation, Data Curation, Writing-Original Draft, Writing-Review & Editing, Visualization Supervision, Project Administration, Funding Acquisition.

## Declaration of Competing Interest

The authors declare the following financial interests/personal relationships which may be considered as potential competing interests: Paul E Croarkin reports financial support was provided by National Institute of Mental Health. Paul E Croarkin reports a relationship with Neuronetics that includes: funding grants. Paul E. Croarkin reports a relationship with MagVenture Inc that includes: funding grants. Paul E. Croarkin reports a relationship with Innosphere that includes: funding grants. Paul E. Croarkin reports a relationship with Mind Medicine Inc. that includes: consulting or advisory. Paul E. Croarkin reports a relationship with Mayo Clinic in Rochester that includes: employment. Paul E. Croarkin reports a relationship with Journal of Child and Adolescent Psychopharmacology that includes: employment. The other authors have no disclosures.
